# All-*trans *retinoic acid induces COX-2 and prostaglandin E_2 _synthesis in SH-SY5Y human neuroblastoma cells: involvement of retinoic acid receptors and extracellular-regulated kinase 1/2

**DOI:** 10.1186/1742-2094-4-1

**Published:** 2007-01-04

**Authors:** Matilde Alique, Juan F Herrero, Francisco Javier Lucio-Cazana

**Affiliations:** 1 Facultad de Medicina, Departamento de Fisiología, Campus Universitario, Universidad de Alcalá, 28871 Madrid, Spain

## Abstract

**Background:**

Our recent results show that all-*trans *retinoic acid (ATRA), an active metabolite of vitamin A, induces COX-dependent hyperalgesia and allodynia in rats. This effect was mediated by retinoic acid receptors (RARs) and was associated with increased COX-2 expression in the spinal cord. Since ATRA also up-regulated COX-2 expression in SH-SY5Y human neuroblastoma cells, the current study was undertaken to analyze in these cells the mechanism through which ATRA increases COX activity.

**Methods:**

Cultured SH-SY5Y neuroblastoma cells were treated with ATRA. COX expression and kinase activity were analyzed by western blot. Transcriptional mechanisms were analyzed by RT-PCR and promoter assays. Pharmacological inhibitors of kinase activity and pan-antagonists of RAR or RXR were used to assess the relevance of these signaling pathways. Production of prostaglandin E_2 _(PGE_2_) was quantified by enzyme immunoabsorbent assay. Statistical significance between individual groups was tested using the non-parametric unpaired Mann-Whitney U test.

**Results:**

ATRA induced a significant increase of COX-2 expression in a dose- and time-dependent manner in SH-SY5Y human neuroblastoma cells, while COX-1 expression remained unchanged. Morphological features of differentiation were not observed in ATRA-treated cells. Up-regulation of COX-2 protein expression was followed by increased production of PGE_2_. ATRA also up-regulated COX-2 mRNA expression and increased the activity of a human COX-2 promoter construct. We next explored the participation of RARs and mitogen-activated peptide kinases (MAPK). Pre-incubation of SH-SY5Y human neuroblastoma cells with either RAR-pan-antagonist LE540 or MAP kinase kinase 1 (MEK-1) inhibitor PD98059 resulted in the abolition of ATRA-induced COX-2 promoter activity, COX-2 protein expression and PGE_2 _production whereas the retinoid X receptor pan-antagonist HX531, the p38 MAPK inhibitor SB203580 or the c-Jun kinase inhibitor SP600125 did not have any effect. The increase in RAR-β expression and extracellular-regulated kinase 1/2(ERK1/2) phosphorylation in ATRA-incubated cells suggested that RARs and ERK1/2 were in fact activated by ATRA in SH-SY5Y human neuroblastoma cells.

**Conclusion:**

These results highlight the importance of RAR-dependent and kinase-dependent mechanisms for ATRA-induced COX-2 expression and activity.

## Background

The initiation and maintenance of central sensitization involve numerous neuromediators. The expression of cyclooxygenase-2 (COX-2), for example, is enhanced rapidly in the spinal cord during sensitization, along with the production of prostaglandins like prostaglandin E_2 _(PGE_2_) [1]. Interleukin-1β (IL-1β) is also up-regulated following inflammation and induces up-regulation of COX-2 in the spinal cord [1]. The mechanisms underlying the up-regulation of COX-2 are not known. Retinoids might be one of these unidentified systems [2].

Biologically active retinoids, a family of vitamin A metabolites or analogues, such as all-*trans *retinoic acid (ATRA) [3], play an essential activity in the embryological development of several tissues and organs [4], including the brain and the spinal cord [3,5]. Retinoids are also present in the brain and spinal cord of adult rats and mice [6,7] and are involved in functions such as spatial learning and memory [8,9]. ATRA is the carboxylic acid form of vitamin A and is considered its major metabolite.

Physiological retinoids are characterized by their capacity to bind and activate retinoid nuclear receptors, including retinoic acid receptors (RARs) and/or retinoid X receptors (RXRs), each having three isotypes, α, β and γ. RARs and RXRs have been identified in numerous tissues including spinal cord [10]. The actions of ATRA are generally mediated by binding to RARs, which act as ligand-regulated transcription factors by binding as hetetodimers with the RXRs to ATRA response elements (RAREs) located in regulatory regions of target genes [11]. Other signalling pathways may also mediate the effects of retinoids and, in the context of the present work, it is particularly relevant the fact that ATRA enhances extracellular-regulated kinase 1/2 (ERK1/2) phosphorylation [12-15], since we have recently found ATRA in human mesangial cells that ERK1/2 plays a key role in the up-regulation of COX-2 by ATRA [16].

In a previous work carried out in our laboratory [2] we observed that rats with inflammation treated with ATRA p.o. showed a more intense development of allodynia and hyperalgesia than control rats. Also, the recovery to baseline was slower in animals treated with ATRA. We also observed that ATRA up-regulated COX-2 expression in SH-SY5Y human neuroblastoma cells, a clonal derivative of the human neuroblastoma SK-N-SH cell line that expresses RARs and RXRs [17,18], and in whole spinal cord of animals treated with ATRA. Further studies [19] indicated that oral treatment with ATRA in normal rats induces a sensitization-like effect on spinal cord neuronal responses similar to that observed in animals with inflammation, and might explain the enhancement of allodynia and hyperalgesia observed in previously published behavioral experiments. The mechanism of action involved an over-expression of COX-2, but not COX-1, in the lumbar spinal cord [19]. When ATRA was administered intrathecally, the sensitization-like effect was inhibited by a RAR-pan-antagonist and associated with a modulation of COX-2 and IL-1 activities [20].

The current study was undertaken to analyze in SH-SY5Y human neuroblastoma cells the mechanism through which ATRA increases COX activity. Preliminary results have been published in abstract form [21].

## Materials and methods

### Drugs and other reagents

The RARs pan-antagonist ATRA (all *trans*-retinoic acid) was purchased from Sigma (St. Louis, MO). The selective RAR pan-antagonist (LE540) and RXR pan-antagonist (HX531) were kindly provided by Dr. Kagechika (School of Biomedical Science, Tokyo Medical and Dental University, Tokyo, Japan). The mitogen-activated protein kinase (MAPK) inhibitors: PD98059 (MAPK kinase (MKK1) inhibitor), SB203580 (p38 MAPK inhibitor) and SP600125 (JNK MAPK inhibitor) were purchased from Calbiochem (La Jolla, CA). Interleukin-1β was purchased from Roche (Indianapolis, IN). All reagents were prepared in DMSO so that the final concentration was < 0.1%, except ATRA, which was dissolved in ethanol, and interleukin-1β, which was dissolved in sterile water. The human COX-2 luciferase reporter construct phPES2 containing the promoter fragment -327 to +59 [22] was a gift from Dr. Hiroyasu Inoue (Nara Women's University, Nara, Japan). Primary antibodies against COX-1, COX-2, RAR-β and total ERK2 were purchased from Santa Cruz Biotechnology (Santa Cruz, CA). Antibody against phosphorylated form of ERK1/2 was purchased from Cell Signaling Technology (Danvers, MA) and an anti-α-actin antibody was from Sigma Chemical Co (St. Louis, MO). All antibodies were used at 1:1000 dilution.

### Cell culture

The SH-SY5Y human neuroblastoma cell line (N-type cells, derived from the parental cell line SK-N-SH; Biedler et al. 1973) was obtained from American Type Culture Collection (Cat #: CRL-2266; ATCC, Manasas, VA). The culture medium was DMEM (Invitrogen, CA) supplemented with 10% fetal bovine serum (FBS), 20 mM L-glutamine and antibiotics (penicillin 100 U/ml and streptomycin 100 μg/ml). Confluent cultures were used and they were made quiescent when appropriate by a 24 h incubation with medium supplemented with 0.5% FBS. ATRA treatment did not induce morphological features of differentiation.

### Transient transfection and luciferase assay

Cells, 3.5 × 10^5 ^per well, were plated in 6-well plates 24 h before transfection. The cells in every well were then incubated 8 h at 37°C with 2 ml Opti-MEM (Invitrogen, CA) cointaining complexes of 5 μg lipofectAMINE (Invitrogen, CA), 1 μg human COX-2 reporter and 0.1 μg renilla luciferase reporter as an internal control. Transfected cells were next incubated with complete growth medium for 16 h and then treated with either ATRA (10 μM, 24 h) or with IL-1β (10 ng/ml, 24 h), and in other experiments pre-incubated with for 1 h with either 2.5 μM LE540 or 50 μM PD98059 and then with ATRA (10 μM, 24 h). Finally, firefly luciferase activity of the COX-2 reporter was measured with a Lumat LB9506 luminometer (Berthold Technologies, Herts, UK) and normalized against the renilla luciferase activity by using the dual-luciferase reporter assay system (Promega, Madison, WI). The experiments were performed in triplicate and repeated four times (for statistical purposes n = 4).

### Western blot analysis

Cells were homogenized in a solution containing 150 mM NaCl, 10 mM Tris-HCl (pH 7.4), 5 mM EDTA, 1% deoxycholic acid, 0.1% SDS, 1% Triton X-100 and protease inhibitors 1 mM phenyl-methyl-sulfonyl-fluoride, 10 μ/ml aprotinin, 2 μg/ml leupeptin and the phosphatase inhibitir 0.2 mM NaVO_4_. Cell proteins (30–40 μg) were run in 8–10% SDS-polyacrilamide gels, transferred onto a nitrocellulose membrane (Trans-Blot Transfer Medium, Bio-Rad, CA) and incubated overnight at 4°C with antibodies recognizing specifically COX-1, COX-2, RAR-β, P-ERK1/2 as previously described [2]. This incubation was followed by a second incubation with peroxidase-conjugated secondary antibody and immunoreactive products were detected by chemiluminiscence using the ECL Western Blotting Detection Reagents (Amersham Biosciences, UK) following the protocol provided by the manufacturer. As a loading control, blots probed with anti-COX-1, anti-COX-2 and anti-RAR-β were subsequently re-probed with anti-α-actin, whereas blots probed with anti-P-ERK1/2 were re-probed with anti-total ERK2. Each experiment was performed at least three times.

### RT-PCR analysis of COX-2 expression

Total RNA was extracted using the TriPure isolation reagent (Roche Diagnostics, GmbH, Mannheim, Germany) according to the manufacturer's instructions and spectrophotometrically quantified. The RT-PCR reaction was performed with the cMaster RTplusPCR system (Eppendorf AG, Hamburg, Germany) with specific primers for human COX-2 purchased to Ambion (Austin, TX) [(F) 5'-CATTCTTTGCCCAGCACTTCAC-3'; (R) GACCAGGCACCAGACCAAAGAC; Accession number: D28235]. Modified 18S primers (QuantumRNA 18S Internal Standards; Ambion) were used for 18S coamplification, as constitutive controls.

The reaction mixture was incubated for 60 min at 42°C and 2 min at 94°C, followed by 35 cycles of 30 sec at 94°C, 30 sec at 59°C and 30 sec at 72°C, with a final extension of 5 min at 72°C. Preliminary experiments established that these conditions provided a linear cDNA amplification. PCR products were separated on 2% agarose gels, and bands were visualized by ethidium bromide staining. Each experiment was performed three times.

### Determination of PGE_2 _formation

**Figure 1:**
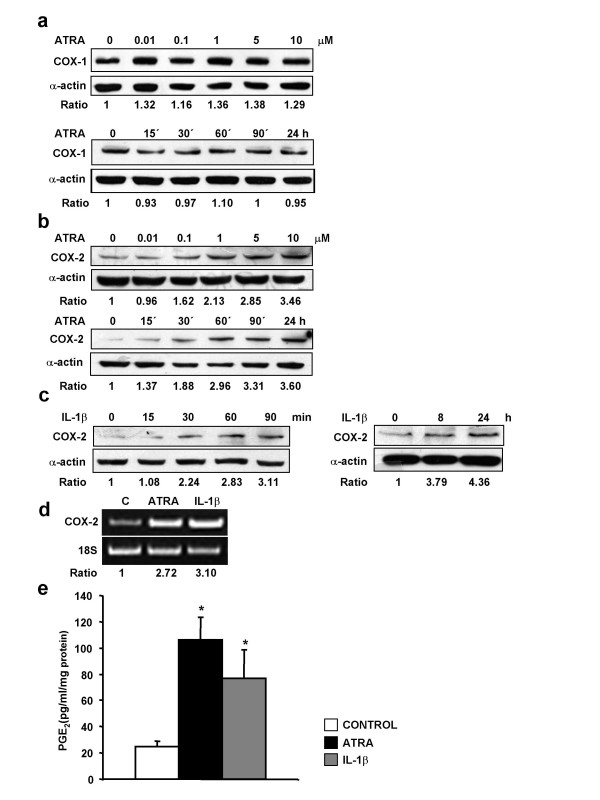
***ATRA up-regulates COX-2 expression and increases PGE*_2 _*in SH-SY5Y human neuroblastoma cells***. (a,b) Expression of COX-1 and COX-2 proteins was analyzed by western blot in SH-SY5Y human neuroblastoma cells incubated for 24 h with the indicated concentrations of ATRA or incubated with 10 μM ATRA for the indicated times. (c) For comparison, expression of COX-2 protein was also analyzed in cells incubated with the inflammatory cytokine interleukin-1 β (IL-1β,10 ng/ml). (d) Expression of COX-2 mRNA, analyzed by semiquantitative RT-PCR in cells incubated for 24 h with 10 μM ATRA or 10 ng/ml IL-1β. (e) PGE_2 _production in SH-SY5Y human neuroblastoma cells incubated for 24 h with 10 μM ATRA or 10 ng/ml IL-1β. PGE_2 _in the medium was determined in triplicate in four separate experiments (for statistical purposes n = 4; * P < 0.01 vs control). (a,b,c,d) Each photograph represents at least three repeated experiments. Equal protein or mRNA loading were confirmed by probing with an anti-α-actin antibody or by co-amplification of 18 S RNA, respectively Normalized density ratio of COX-2 over either α-actin or 18 S RNA is indicated for each band.

The cultured medium of SH-SY5Y human neuroblastoma cells growth in 6-plates and treated as described in the legends of Figures 1e and 3b was collected and diluted 2 times. PGE_2 _concentrations in the medium were determined in triplicate using a commercially available enzyme immunoabsorbent assay (EIA) kit (Cayman Chemical Company, Ann Arbor, MI) following the manufacturer's protocol. The assay was performed in a total volume of 150 μl, with the following components being added in 50 μl volumes: standards or biological samples, enzymatic tracer and specific antiserum. After overnight incubation at 4°C, the plates were washed, and 200 μl Ellman's reagent was added into each well. After 1–3 h, the absorbance at 414 nm of each well was measured. A standard curve, with values ranging from 50 to 0.39 pg/ml, was used to evaluate the concentrations. The reliable limit of quantification for PGE_2 _was 15 pg/ml, and the coefficient of variation was less than 14% within the calibration range (15–1000 pg/ml). Results were calculated by using the nonlinear regression of a four-parameter logistic model. Each experiment was performed four times (for statistical purposes n = 4).

### Data analysis and statistical procedures

All values are presented as mean ± standard error of the mean (s.e.m). All experiments were repeated a minimum of three times. Statistical significance between individual groups was tested using the non-parametric unpaired Mann-Whitney U test. A P value of < 0.05 was considered significant.

## Results

### ATRA up-regulates the expression of COX-2 protein and COX-2 mRNA as well as the production of PGE_2 _in SH-SY5Y human neuroblastoma cells

The effect of ATRA on the levels of COX-1 and COX-2 proteins was examined in typical dose-response and time-course experiments. Serum-deprived cultured SH-SY5Y human neuroblastoma cells constitutively expressed both COX-1 and COX-2 in the absence of stimulation (Figure 1a, b). COX-1 expression was not modified by incubation with the retinoid (Figure 1a) whereas ATRA treatment up-regulated COX-2 expression in dose- and time-dependently manner. As shown in Figure 1b, the COX-2 protein levels increased early after the treatment with 10 μM ATRA, modestly after 30 minutes and markedly after 24 hours. Treatment with the pro-inflammatory mediator IL-1β (10 ng/ml) rendered similar results (Figure 1c). Equal protein loading was confirmed by re-probing with an anti-α-actin antibody.

We next examined the effect of ATRA on the expression of COX-2 mRNA. Serum-deprived human SH-SY5Y human neuroblastoma cells were treated with or without ATRA for 24 hours, and semiquantitative RT-PCR was performed. Basal COX-2 mRNA expression in SH-SY5Y human neuroblastoma cells was up-regulated by treatment with ATRA (Figure 1d) to a similar extent than that found in cells treated with IL-1β (10 ng/ml).

We finally confirmed that the up-regulation of COX-2 protein expression was followed by increased production of PGE_2_. Basal release of PGE_2 _over 24 h was substantially increased after incubation with 10 μM ATRA (Figure 1e). The increase in PGE_2 _production was similar to that found when SH-SY5Y human neuroblastoma cells were incubated with IL-1β (10 ng/ml).

### ATRA increases the activity of the human COX-2 promoter and its effect is inhibited by RAR-pan-antagonist LE540 or MEK-1 inhibitor PD98059

**Figure 2:**
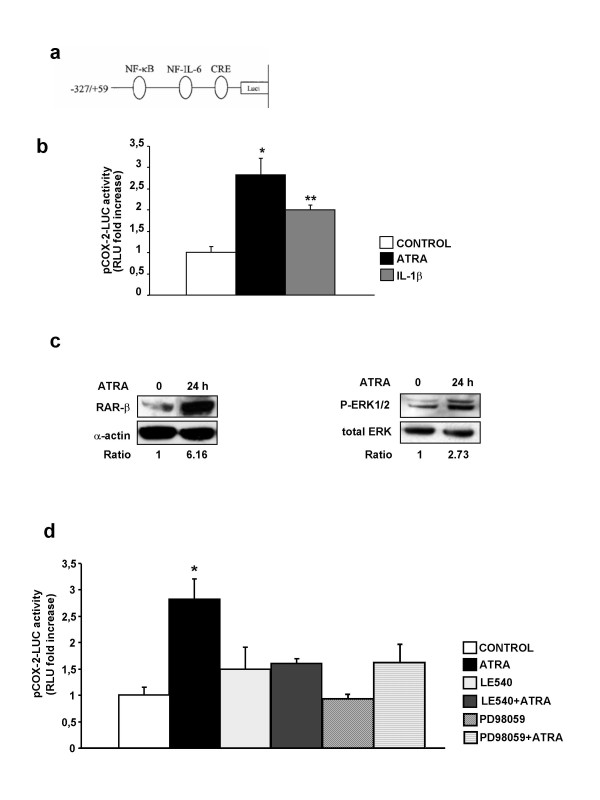
***ATRA increases the activity of the human COX-2 promoter and its effect is inhibited by RAR-pan-antagonist LE540 or MEK-1 inhibitor PD98059***. (a) Schematic of the COX-2 human promoter construct phPES2 containing the promoter fragment -327 to +59.. (b) ATRA (10 μM, 24 h) increases the activity of the human COX-2 gene promoter transfected in SH-SY5Y human neuroblastoma cells. For comparison, the effect of IL-1β (10 ng/ml, 24 h) is also shown. COX-2 promoter activity was determined in triplicate in four separate experiments (for statistical purposes n = 4; *P < 0.01 vs other groups; **P < 0.01 vs control). (c) ATRA (10 μM, 24 h) increases the expression of RAR-β (left) and induces ERK1/2 phosphorylation (right). Normalized density ratio of either RAR-β or ERK1/2 over α-actin is indicated for each band. Each photograph represents at least three repeated experiments. (d) Inhibition of ATRA-induced COX-2 promoter activity by the RAR-pan-antagonist LE540 and the MEK-1 inhibitor PD98059. Transiently transfected cells were pre-incubated for 1 h with either 2.5 μM LE540 or 50 μM PD98059 and then with ATRA (10 μM, 24 h). COX-2 promoter activity was measured in triplicate in four separate experiments (for statistical purposes n = 4) (*P < 0.01 vs other groups)

To examine whether ATRA can induce transcription from the COX-2 promoter, we used phPES2 (-327/+59; Figure 2a), a plasmid that expresses firefly luciferase (LUC) under the control of the human COX-2 gene promoter (-327/+59). Transient transfection assay showed that ATRA increased the activity of the human COX-2 gene promoter than that observed with IL-1β treatment (Figure 2b).

**Figure 3:**
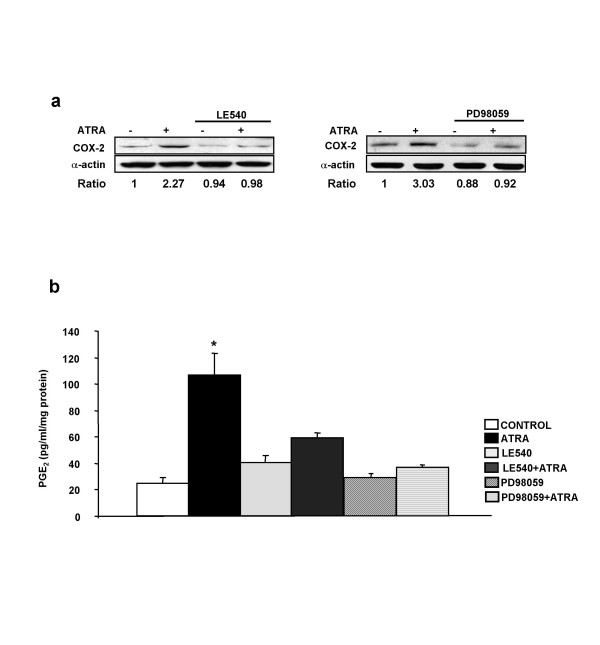
***ATRA-induced COX-2 protein expression and PGE*_2 _*production are inhibited by RAR pan-antagonist LE540 or MEK-1 inhibitor PD98059***. (a) Western blot analysis of the expression of COX-2 protein in SH-SY5Y human neuroblastoma cells pre-incubated for 1 h with either RAR pan-antagonist LE540 2.5 μM (left) or 50 μM MEK-1 inhibitor PD98059 (right) and then incubated with ATRA (10 μM, 24 h). Equal protein loading was confirmed by probing with an anti-α-actin antibody. Normalized density ratio of COX-2 over α-actin is indicated for each band. Each experiment represents at least three repeated experiments. (b) PGE_2 _production in SH-SY5Y human neuroblastoma cells incubated for 24 hours with ATRA 10 μM in cells and pre-treated for 1 hour with either RAR pan-antagonist LE540 2.5 μM or 50 μM MEK-1 inhibitor PD98059. PGE_2 _in the medium was determined in triplicate in four separate experiments (for statistical purposes n = 4) (*P < 0.01 vs other groups)

The actions of ATRA are generally mediated by binding to RARs and we have recently found that ATRA-induces spinal cord sensitization through a mechanism involving RAR-mediated COX-2 up-regulation [16]. Nevertheless, ATRA may also have RAR-independent effects being ERK1/2-dependent mechanisms particularly important for ATRA-induced COX-2 up-regulation in renal mesangial cells [23]. Activation of RAR-dependent mechanisms by ATRA was indirectly assessed in SH-SY5Y human neuroblastoma cells by western blot analysis of ATRA-induced RAR-β expression, since this receptor contains a RARE in its promoter [23] (Figure 2c, left). In turn, ERK1/2 phosphorylation by incubation with ATRA was confirmed by western blot analysis (Figure 2c, right). We therefore designed experiments involving pharmacologic inhibitors to analyze the specific contribution of RAR- and/or ERK1/2-dependent mechanisms to ATRA-induced COX-2 promoter activity. Preincubation for 1 h with either the RAR pan-antagonist LE540 or the selective inhibitor of MEK-1 PD98059 abolished ATRA-induced COX-2 promoter activity (Figure 2d) whereas the RXR pan-antagonist HX531, the p38 MAPK inhibitor SB203580 or the c-Jun kinase inhibitor SP600125 did not have any effect (data not shown).

### ATRA-induced COX-2 protein expression and PGE_2 _production are inhibited by RAR pan-antagonist LE540 or MEK-1 inhibitor PD98059

To confirm the relevance of the findings described above, we studied the effect of LE540 and PD98059 on ATRA-induced COX-2 protein expression and PGE_2 _production. Cells were treated for 1 hour with the inhibitors and then they were stimulated by ATRA for 24 h. The results showed that ATRA-induced COX-2 protein expression and PGE_2 _production were both inhibited by either RAR pan-antagonist LE540 or MEK-1 inhibitor PD98059 (Figure 3a left, 3a right, 3b).

## Discussion

The aim of the present work was to examine the mechanisms involved in ATRA-induced COX-2 up-regulation in human neuroblastoma SH-SY5Y. Our results indicate that ATRA up-regulates COX-2 but not COX-1 expression and that transcriptional mechanisms and ERK1/2 are specifically involved in COX-2 up-regulation. These results are identical to those previously obtained in human [20] and rat [24] renal mesangial cells in culture. Since ATRA also up-regulates COX-2 expression in rat spinal cord [17], in rat kidney [2] and in canine kidney tubular cells MDCK (unpublished observations), our results suggest that ATRA is a main regulator of COX-2 expression in nervous and renal tissues.

Increased activity of the COX-2 promoter resulting in COX-2 mRNA up-regulation (Figures 1d and 2b) is likely involved in COX-2 up-regulation by ATRA, although post-transcriptional mechanisms may also be involved. The transcriptional effects of ATRA are most commonly mediated by binding to nuclear receptors RARs, which normally act as ligand-inducible transcription factors by binding, as heterodimers with the RXRs, to DNA response elements known as retinoic acid response elements [25]. Since there are no retinoic acid-response elements identified in the human COX-*2 *promoter [26], ATRA-activated RAR-RXR heterodimers are not expected to be involved in a direct activation of the COX-2 promoter. In this context, the abolition by RAR pan-antagonist LE540 of ATRA-induced COX-2 promoter activation (Figure 2d) is more likely due to the inhibition of a RAR-dependent effect on an unidentified target. This unknown target of ATRA would, in turn, be responsible for the activation of a signalling pathway leading to the activation of the COX-2 promoter. Previous studies have found contradictory results on the effect of ATRA on COX-2 expression: For instance, ATRA repressed COX-2 promoter activity and COX-2 mRNA expression in several murine lung tumour-derived cell lines, yet it increased promoter activity and COX-2 mRNA expression significantly in another lung tumour-derived cell line [27]. In other studies ATRA did not induce COX-2 in phorbol 12-myristate 13-acetate-differentiated U937 cells [28] whereas it suppressed COX-2 transcription in human mammary epithelial cells [29]. There are also observations showing that, in certain cell lines, RAR and RXR inhibit COX-2 promoter activity [30,31]. Finally, our own studies in renal mesangial cells indicate that RARs are involved in ATRA-induced COX-2 expression in cells cultured from rats [24] but not in cells cultured from human donors [20]. These data indicate that the effect of ATRA on COX-2 expression is likely to be cell-specific.

We and others have reported that ATRA enhances ERK1/2 phosphorylation [14,32-34] and that ERK1/2 plays a key role in the up-regulation of COX-2 by ATRA in human and rat mesangial cells [20,24]. Here we observed that ATRA also induces ERK1/2 phosphorylation in SH-SY5Y human neuroblastoma cells (Figure 2c, right) and we confirmed the key role of ERK1/2 phosphorylation for COX-2 up-regulation by ATRA since treatment with PD098059, the selective inhibitor of mitogen-activated protein kinase kinase 1 (MEK-1), was sufficient to abrogate COX-2 promoter activation, to increase COX-2 protein expression and to increase PGE_2 _production (Figure 2d and Figure 3a right and 3b). The mechanism by which ATRA can cause ERK1/2 activation is still unknown. For neuronal cells, it has been suggested that a subpopulation of classical RAR receptors, localized at or near the cell membrane, could be responsible for ATRA-induced CREB (cyclic AMP-response-element-binding protein) activation through ERK1/2 phosphorylation [14]. We have proposed that such mechanism could also be responsible for ATRA-induced COX-2 up-regulation through ERK1/2 phosphorylation in rat mesangial cells [24]. In summary, the induction of ERK1/2 phosphorylation by ATRA is a RAR-independent key event though which the retinoid increases COX-2 expression and PGE_2 _production in SH-SY5Y human neuroblastoma cells.

Intrathecal administration of ATRA to normal rats enhances nociceptives responses as well as responses to innocuous stimulation through a COX-2 dependent mechanism [20]. We describe here, in SH-SY5Y human neuroblastoma cells, that ATRA induces COX-2 expression resulting in increased PGE_2 _production through a mechanism involving ERK1/2. A confirmation of these data in primary cultures of neuronal cells is needed to support a role in spinal cord sensitization of ATRA-induced PGE_2 _production.

## Conclusion

In conclusion, our results indicate that ATRA induces activity of the COX-2 promoter and synthesis of COX-2 mRNA and COX-2 protein, resulting in increased PGE_2 _production in SH-SY5Y human neuroblastoma cells; and that RARs and ERK1/2 were required for these ATRA effects. This highlights the importance of RAR-dependent and kinase-dependent mechanisms for ATRA-induced COX-2 expression and activity.

## Abbreviations

Cyclooxygenase (COX); All-*trans *retinoic acid (ATRA); Prostaglandins (PGs); Prostaglandin E_2 _(PGE_2_); Extracellular signal-regulated kinase 1/2 (ERK1/2); Interleukin-1β (IL-1β); Mitogen-activated protein kinase (MAPK); Enzyme immunoabsorbent assay (EIA); Retinoic acid receptor (RAR); Retinoid X receptor (RXR); MAP kinase kinase 1(MEK-1); Jun N-terminal kinase, (JNK).

## Competing interests

The author(s) declare that they have no competing interests.

## Authors' contributions

MA carried out all the experiments. JFH participated in the design of the study and performed the statistical analysis. FJLC conceived of the study, and participated in its design and coordination and helped to draft the manuscript. All authors have read and approved the final manuscript.
